# Approximate Analytical Solutions of the Regularized Long Wave Equation Using the Optimal Homotopy Perturbation Method

**DOI:** 10.1155/2014/721865

**Published:** 2014-06-03

**Authors:** Constantin Bota, Bogdan Căruntu

**Affiliations:** Department of Mathematics, Politehnica University of Timişoara, P-ta Victoriei 2, 300006 Timişoara, Romania

## Abstract

The paper presents the optimal homotopy perturbation method, which is a new method to find approximate analytical solutions for nonlinear partial differential equations. Based on the well-known homotopy perturbation method, the optimal homotopy perturbation method presents an accelerated convergence compared to the regular homotopy perturbation method. The applications presented emphasize the high accuracy of the method by means of a comparison with previous results.

## 1. Introduction

A significant part of the natural technological processes and phenomena are usually modelled by means of partial differential equations. Thus it is very important to find solutions of these equations. However, as in many cases the computation of exact solutions is not possible; numerical or approximate solutions must be computed.

In the present paper we present a new approximation method named optimal homotopy perturbation method (OHPM). As the name suggests, the method is based on the homotopy perturbation method [1, 2] and its main feature is an accelerated convergence compared to the regular homotopy perturbation method.

The applications presented show that the approximate solutions obtained by using OHPM requires less iterations in comparison with other iterative methods for approximate solutions of partial differential equations.

## 2. The Optimal Homotopy Perturbation Method

We consider the following problem:

(1) $$\begin{array}{c} L\left(u\left(x,t\right)\right)+N\left(u\left(x,t\right)\right)-f\left(x,t\right)=0,\;\;B\left(u\right)=0. \end{array}$$

Here *L* is a linear operator, *u*(*x*, *t*) is the unknown function, *N* is a nonlinear operator, *f*(*x*, *t*) is a known, given function, and *B* is a boundary operator.

If $\tilde{u}$ is an approximate solution of (1), we evaluate the error obtained by replacing the exact solution *u* with the approximate one $\tilde{u}$ as the remainder:

(2) $$\begin{array}{c} R\left(x,t,\tilde{u}\right)=L\left(\tilde{u}\left(x,t\right)\right)+N\left(\tilde{u}\left(x,t\right)\right)-f\left(x,t\right). \end{array}$$

The first step in applying OHPM is to attach to the problem (1) the family of equations (see [1, 2]):

(3) (1−p)[L(Φ(x,t,p))−f(x,t)]  +p[L(Φ(x,t,p))+N(Φ(x,t,p))−f(x,t)]=0,

where *p* ∈ [0,1] is an embedding parameter and Φ(*x*, *t*, *p*) is an unknown function.

When *p* = 0, Φ(*x*, *t*, 0) = *u*_0_(*x*, *t*) and when *p* = 1, Φ(*x*, *t*, 1) = *u*(*x*, *t*). Thus, as *p* increases from 0 to 1, the solution Φ(*x*, *t*, *p*) varies from *u*_0_(*x*, *t*) to the solution *u*(*x*, *t*), where *u*_0_(*x*, *t*) is obtained from the following:

(4) $$\begin{array}{c} \begin{array}{c} L\left(u_{0}\left(x,t\right)\right)-f\left(x,t\right)=0,\;\;B\left(u_{0}\right)=0. \end{array} \end{array}$$

We consider the following expansion of Φ(*x*, *t*, *p*):

(5) $$\begin{array}{c} \Phi \left(x,t,p\right)=u_{0}\left(x,t\right)+\underset{m\geq 1}{\sum }u_{m}\left(x,t\right)p^{m}. \end{array}$$

Substituting the relation (5) into (3), collecting the same powers of *p*, and equating each coefficient of the powers of *p* with zero we obtain

(6) L(um(x,t))=−Nm−1(u0(x,t),u1(x,t),…,um−1(x,t))m≥1,…,B(um)=0,

where *N*_*i*_, *i* ≥ 0 are the coefficients of *p* ^*i*^ in the nonlinear operator *N*:

(7) N(u(x,t)) =N0(u0(x,t))+pN1(u0(x,t),u1(x,t))  +p2N2(u0(x,t),u1(x,t),u2(x,t))+⋯.

We remark that *u*_*m*_, *m* ≥ 1 are obtained from the linear equations (6), which are easily solved together with the boundary conditions.

We denote *f*_*m*_ = *u*_0_ + *u*_1_ + ⋯+*u*_*m*_.

We consider the set *S*_*m*_ (*m* = 0,1, 2,…) containing the functions *φ*_*m*0_, *φ*_*m*1_, *φ*_*m*2_,…, *φ*_*mn*_*m*__, chosen as linearly independent functions in the vector space of the continuous functions on the real domain *Ω* such that *S*_*m*−1_⊆*S*_*m*_ and *u*_0_ + *u*_1_ + ⋯+*u*_*m*_ is a real linear combination of these functions.

We remark that such a construction is always possible. For example we can choose *S*_*m*_ = {*u*_0_, *u*_1_,…, *u*_*m*_}, *m* = 0,1, 2,…. In this case *φ*_*m*0_ = *u*_0_, *φ*_*m*1_ = *u*_1_, *φ*_*m*2_ = *u*_2_,…, *φ*_*mn*_*m*__ = *u*_*m*_.

**Definition 1**

We call an HP-sequence of the problem (1) a sequence of functions {*s*_*m*_(*x*, *t*)}_*m*∈*N*_ of the form *s*_*m*_(*x*, *t*) = ∑_*k*=0_^*n*_*m*_^ *α*_*m*_^*k*^ *φ*_*mk*_, where *m* ∈ *N*, *α*_*m*_^*k*^ ∈ *R*.

A function of the sequence is called an HP-function of the problem (1).

We call the HP-sequence {*s*_*m*_(*x*, *t*)}_*m*∈*N*_, convergent to the solution of the problem (1) if lim⁡_*m*→*∞*_⁡*R*(*x*, *t*, *s*_*m*_(*x*, *t*)) = 0.

**Definition 2**

We call an *ϵ-approximate* HP-solution of the problem (1) on the real domain *Ω* an HP-function $\tilde{u}$ which satisfies the following condition:

(8) $$\begin{array}{c} \left|R\left(x,t,\tilde{u}\right)\right|<\epsilon \end{array}$$

together with the boundary conditions from (1).

**Definition 3**

We call a* weak δ-approximate HP-solution* of the problem (1) on the real domain *Ω* an HP-function $\tilde{u}$ satisfying the relation $\int_{\Omega }^{}R^{2}\left(x,t,\tilde{u}\right)dx\;dt\leq \delta$, together with the boundary conditions from (1).

We will find a weak *ϵ*-approximate HP-solution of the type $\tilde{u}=\sum_{k=0}^{n_{m}}c_{m}^{k}\varphi_{mk}$ where *m* ≥ 0 and the constants *c*_*m*_^*k*^ are calculated using the following steps.

(i) We substitute the approximate solution $\tilde{u}$ in (1) and obtain the following expression:
  (9) $$\begin{array}{c} R\left(x,t,c_{m}^{k}\right)=R\left(x,t,\tilde{u}\right). \end{array}$$
(ii) We attach to the problem (1) the following real functional:
  (10) $$\begin{array}{c} J\left(c_{m}^{k}\right)=\overset{}{\underset{\Omega }{\int }}R^{2}\left(x,t,c_{m}^{k}\right)dx\;dt, \end{array}$$
 where, by imposing the boundary conditions we can determine *l* ∈ *N*, *l* ≤ *m* such that *c*_0_^*m*^, *c*_1_^*m*^,…, *c*_*l*_^*m*^ are computed as functions of *c*_*l*+1_^*m*^, *c*_*l*+2_^*m*^,…, *c*_*n*_^*m*^.
(iii) We compute the values of ${\tilde{c}}_{l+1}^{m},{\tilde{c}}_{l+2}^{m},\ldots ,{\tilde{c}}_{n}^{m}$ as the values which give the minimum of the functional (10) and the values of ${\tilde{c}}_{0}^{m},{\tilde{c}}_{1}^{m},\ldots ,{\tilde{c}}_{l}^{m}$ again as functions of ${\tilde{c}}_{l+1}^{m},{\tilde{c}}_{l+2}^{m},\ldots ,{\tilde{c}}_{n}^{m}$ by using the boundary conditions.
(iv) Using the constants ${\tilde{c}}_{0}^{m},\ldots ,{\tilde{c}}_{n}^{m}$ thus determined, we consider the HP-sequence
  (11) $$\begin{array}{c} s_{m}\left(x,t\right)=\overset{n_{m}}{\underset{k=0}{\sum }}{\tilde{c}}_{m}^{k}\varphi_{mk}. \end{array}$$

The following convergence theorem holds.

**Theorem 4**

The HP-sequence *s*_*m*_(*x*, *t*) from (11) satisfies the following property:

(12) $$\begin{array}{c} \underset{m\to \infty }{\lim}\overset{}{\underset{\Omega }{\int }}R^{2}\left(x,t,s_{m}\left(x,t\right)\right)dx\;dt=0. \end{array}$$

Moreover, ∀*ϵ* > 0, ∃*m*_0_ ∈ *N* such that ∀*m* ∈ *N*, *m* > *m*_0_ it follows that *s*_*m*_(*t*) is a weak *ϵ*-approximate HP-solution of the problem (1).

**Proof**

Based on the way the HP-function *s*_*m*_(*x*, *t*) is computed, the following inequality holds:

(13) 0≤∫ΩR2(x,t,sm(x,t))dx dt≤∫ΩR2(t,fm(x,t))dx dt, ∀m∈N.

It follows that

(14) 0≤lim⁡m→∞∫ΩR2(x,t,sm(x,t))dx dt≤lim⁡m→∞∫ΩR2(x,t,fm(x,t))dx dt=0, ∀m∈N.

We obtain

(15) $$\begin{array}{c} \underset{m\to \infty }{\lim}\overset{}{\underset{\Omega }{\int }}R^{2}\left(x,t,s_{m}\left(x,t\right)\right)dx\;dt=0. \end{array}$$

From this limit we obtain that ∀*ϵ* > 0, ∃*m*_0_ ∈ *N* such that ∀*m* ∈ *N*, *m* > *m*_0_ it follows that *s*_*m*_(*x*, *t*) is a weak *ϵ*-approximate HP-solution of the problem (1).

**Remark 5**

Any *ϵ*-approximate HP-solution of the problem (1) is also a weak approximate HP-solution, but the opposite is not always true. It follows that the set of weak approximate HP-solutions of the problem (1) also contains the approximate HP-solutions of the problem.

Taking into account the above remark, in order to find *ϵ*-approximate HP-solutions of the problem (1) by the OHPM method we will first determine weak approximate HP-solutions, $\tilde{u}$. If $|R(x,t,\tilde{u})|<\epsilon$ then $\tilde{u}$ is also an *ϵ*-approximate HP-solution of the problem.

## 3. Applications

In this section we apply OHPM to find approximate analytical solutions for the regularized long wave (RLW) equation.

The RLW equation is a nonlinear evolution equation. These kind of equations are frequently used to model a variety of physical phenomena such as ion-acoustic waves in plasma, magnetohydrodynamics waves in plasma, longitudinal dispersive waves in elastic rods, pressure waves in liquid gas bubble mixtures, and rotating flow down a tube.

The RLW equation was introduced in [3] where it was used to describe the behaviour of the undular bore.

For some restricted initial and boundary conditions, exact analytical solutions for the RLW equation were computed (see, e.g., [4]). However, in most cases it is not possible to find such exact analytical solutions and usually numerical methods are used. Among the numerical methods recently employed for RLW-type equations we mention finite difference methods [5–8], multistep mixed finite element methods [9], the method of lines [10], and meshless finite-point methods [11].

Taking into account the usefulness of analytical solutions versus numerical ones, various approximation methods were also employed to find approximate analytical solutions for various RLW-type equations, such as the homotopy perturbation method [12], the variational iteration method [12], the homotopy asymptotic method [13, 14], and the Riccati expansion method [15].

In the following, for two test problems presented in [12], we compare solutions obtained by using OHPM with previous results obtained by using the homotopy perturbation method and the variational iteration method.

### 3.1. Application 1

Our first application is the following RLW problem [12]:

(16) $$\begin{array}{c} u_{t}-u_{xxt}+{\left(\frac{u^{2}}{2}\right)}_{x}=0,\\ u\left(x,0\right)=x. \end{array}$$

In [12] approximate solutions of (16) are computed using the homotopy perturbation method (HPM) and the variational iteration method (VIM).

The exact solution of this problem is *u*_*e*_(*x*, *t*) = *x*/(*t* + 1).

The fifth order solution computed in [12] by using the variational iteration method is

(17) uVIM(x,t) =x·(−t31109876902975+t303544416225−t29236294415+13t28315059220−2t276751269+t26595350−5309t25675126900+16927t24540101520−2447t2322504230+557t221666980−207509t21225042300+16511t207144200−162179t1930541455+2588t18229635−1080013t1748580560+43363t161058400−63283t15893025+1019t148820−13141t1373710+17779t1268040−1477t114050+27523t1056700−3497t95670+943t81260−13t715+43t645−t5+t4−t3+t2−t+1).

The fifth order solution computed in [12] by using the homotopy perturbation method is of the form (5)

(18) uHPM(x,t) =x·(−1382t11155925−1382t1014175−326t9567−626t8315−1303t7315−199t645−t5+t4−t3+t2−t+1).

Using OHPM, the following steps are performed.

- Choosing the same homotopy (3) as used in [12] we obtain the same solutions:
   
*u*_0_(*x*, *t*) = *x* · (*t* + 1)
   
*u*_1_(*x*, *t*) = −*x* · *t* · (2 + *t* + *t* ^2^/3)
   
*u*_2_(*x*, *t*) = 2 · *x* · *t* ^2^ · (15 + 15 · *t* + 5 · *t* ^2^ + *t* ^3^)/15.
- It follows that we obtain the sets *S*_0_ = {*x*, *x* · *t*}, *S*_1_ = {*x* · *t*, *x* · *t* ^2^, *x* · *t* ^3^}, *S*_2_ = {*x* · *t* ^2^, *x* · *t* ^3^, *x* · *t* ^4^, *x* · *t* ^5^}.
- We will compute a second order approximate solution, by taking into account the terms from *S*_0_, *S*_1_, and *S*_2_ and we will compare this solution with the fifth order solutions from [12]. Our second order approximate solution will have the expression *u*_OHPM_(*x*, *t*) = *c*_0_ · *x* + *c*_1_ · *x* · *t* + *c*_2_ · *x* · *t* ^2^ + *c*_3_ · *x* · *t* ^3^ + *c*_4_ · *x* · *t* ^4^ + *c*_5_ · *x* · *t* ^5^.
- Imposing the boundary condition *u*_OHPM_(*x*, 0) = *x* we obtain *c*_0_ = 1.
- Replacing this expression of *c*_0_ in the expression of *u*_OHPM_ we obtain the following:
- *u*_OHPM_(*x*, *t*) = *x* + *c*_1_ · *x* · *t* + *c*_2_ · *x* · *t* ^2^ + *c*_3_ · *x* · *t* ^3^ + *c*_4_ · *x* · *t* ^4^ + *c*_5_ · *x* · *t* ^5^.
- We introduce *u*_OHPM_ in the remainder *R* given by (2) and (9) and we compute the functional *J*(*c*_1_, *c*_2_, *c*_3_, *c*_4_, *c*_5_) of (10).
- We remark that while the expression of the functional is too long to be included here, the computation is simple and straightforward using a dedicated mathematical software (we used the Wolfram Mathematica 9 software).
- We compute the minimum of the functional *J* and, by replacing the corresponding values of the parameters *c*_1_, *c*_2_, *c*_3_, *c*_4_, *c*_5_, we obtain the following second order approximation:
- ${\tilde{u}}_{\text{OHPM}}(x,t)=-0.109895t^{5}x+0.434798t^{4}x-0.789112t^{3}x+0.961938t^{2}x-0.997729tx+x$.

**Figure 1:**
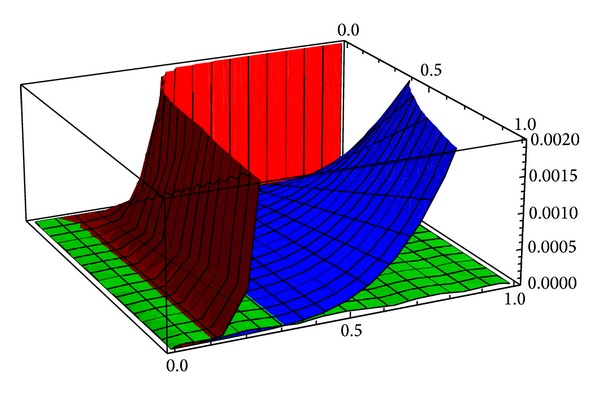
The absolute differences corresponding to the HPM solution (red surface), VIM solution (blue surface), and OHPM solution (green surface) for problem (16).

Figure 1 presents the comparison of the absolute errors (computed as the absolute values of the differences between the exact solutions and the approximate solutions) corresponding to the fifth order approximation obtained by using HPM (red surface), to the fifth order approximation obtained by using VIM (blue surface) and to the second order approximation obtained by OHPM (green surface).

Table 1 presents the same comparison for several values of *x* and *t*.

It is easy to see that, overall, the approximations obtained by using OHPM are much more accurate than the ones previously computed by using HPM and VIM. Moreover, our approximate solutions are not only more accurate but also, at the same time, present a much simpler expression since they are second order approximate solutions while the previous ones are fifth order approximate solutions.

### 3.2. Application 2

Our second application is the RLW problem (also from [12]):

(19) $$\begin{array}{c} u_{t}-u_{xxxx}=0,\\ u\left(x,0\right)=\sin\left(x\right). \end{array}$$

Again in [12] approximate solutions of (16) are computed using the homotopy perturbation method (HPM) and the variational iteration method (VIM).

The exact solution of this problem is *u*_*e*_(*x*, *t*) = *e* ^−*t*^sin(*x*).

The fourth order solution computed in [12] by using the variational iteration method is *u*_VIM_(*x*, *t*) = (1/24)(*t* ^4^ − 4*t* ^3^ + 12*t* ^2^ − 24*t* + 24)sin(*x*).

The third order solution computed in [12] by using the homotopy perturbation method is of the form (5) *u*_HPM_(*x*, *t*) = −(1/24)(*t* ^4^ + 4*t* ^3^ − 12*t* ^2^ + 24*t* − 24)sin(*x*).

Using OHPM, the following steps are performed.

- Choosing the same homotopy (3) as used in [12] we obtain the same solutions:
   
*u*_0_(*x*, *t*) = (*t* + 1) · sin(*x*)
   
*u*_1_(*x*, *t*) = (1/2) · *t* · (*t* + 4)·(−sin(*x*))
   
*u*_2_(*x*, *t*) = (1/6) · *t* ^2^ · (*t* + 6) · sin(*x*).
- It follows that we obtain the sets *S*_0_ = {sin(*x*), sin(*x*) · *t*}, *S*_1_ = {sin(*x*) · *t*, sin(*x*) · *t* ^2^}, *S*_2_ = {sin(*x*) · *t* ^2^, sin(*x*) · *t* ^3^}.
- Hence we will compute a second order approximate solution of the following form:
- *u*_OHPM_(*x*, *t*) = *c*_0_ · sin(*x*) + *c*_1_ · sin(*x*) · *t* + *c*_2_ · sin(*x*) · *t* ^2^ + *c*_3_ · sin(*x*) · *t* ^3^.
- Imposing the boundary condition *u*_OHPM_(*x*, 0) = *x* we obtain *c*_0_ = 1.
- Replacing this expression of *c*_0_ in the expression of *u*_OHPM_ we obtain the following:
- *u*_OHPM_(*x*, *t*) = sin(*x*) + *c*_1_ · sin(*x*) · *t* + *c*_2_ · sin(*x*) · *t* ^2^ + *c*_3_ · sin(*x*) · *t* ^3^.
- We introduce *u*_OHPM_ in the remainder *R* given by (2) and (9) and we compute the functional *J*(*c*_1_, *c*_2_, *c*_3_) of (10).
- We compute the minimum of the functional *J* and, by replacing the corresponding values of the parameters *c*_1_, *c*_2_, *c*_3_, we obtain the following second order approximation:
- ${\tilde{u}}_{\text{OHPM}}(x,t)=-0.102902t^{3}\sin(x)+0.465235t^{2}\sin(x)-0.994455t\sin(x)+\sin(x)$.

**Figure 2:**
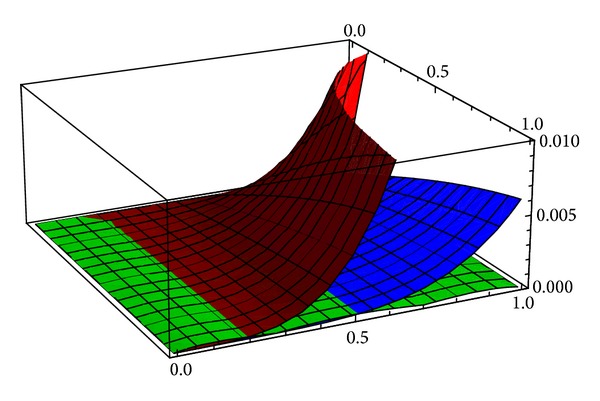
The absolute differences corresponding to the HPM solution (red surface), VIM solution (blue surface), and OHPM solution (green surface) for problem (19).

Figure 2 presents the comparison of the absolute errors corresponding to the third order approximation obtained by using HPM (red surface), to the fourth order approximation obtained by using VIM (blue surface), and to the second order approximation obtained by OHPM (green surface).

Table 2 presents the same comparison for several values of *x* and *t*.

Again, overall, the approximations obtained by using OHPM are more accurate than the ones previously computed by using HPM and VIM while, at the same time, they present a much simpler expression.

## 4. Conclusions

In the present paper the new optimal homotopy perturbation method is introduced as a straightforward and efficient method to compute approximate solutions for nonlinear partial differential equations.

The optimal homotopy perturbation method has an accelerated convergence compared to the regular homotopy perturbation method, fact proved by the included applications. The method is a powerful one since not only were we capable to find more accurate approximations, but also the approximations computed consist of fewer terms than the previous solutions.

## Figures and Tables

**Table 1:** The absolute differences corresponding to the HPM solution (red surface), VIM solution (blue surface), and OHPM solution (green surface) for problem (16).

	HPM	VIM	OHPM
*x* = *t* = 0	0	0	0
*x* = *t* = 0.2	7.894 10^−5^	3.256 10^−7^	1.081 10^−5^
*x* = *t* = 0.4	1.171 10^−2^	2.555 10^−5^	1.398 10^−5^
*x* = *t* = 0.6	2.346 10^−1^	2.819 10^−4^	9.787 10^−6^
*x* = *t* = 0.8	2.075	1.420 10^−3^	3.280 10^−5^
*x* = *t* = 1	1.172 10^1^	4.700 10^−3^	2.408 10^−7^

**Table 2:** The absolute differences corresponding to the HPM solution (red surface), VIM solution (blue surface), and OHPM solution (green surface) for problem (19).

	HPM	VIM	OHPM
*x* = *t* = 0	0	0	0
*x* = *t* = 0.2	2.598 10^−5^	5.126 10^−7^	3.268 10^−5^
*x* = *t* = 0.4	7.996 10^−4^	3.113 10^−5^	9.737 10^−5^
*x* = *t* = 0.6	5.766 10^−3^	3.322 10^−4^	1.279 10^−4^
*x* = *t* = 0.8	2.276 10^−2^	1.725 10^−3^	1.233 10^−4^
*x* = *t* = 1	6.413 10^2^	5.992 10^−3^	9.511 10^−7^
